# Addition of Various Cellulosic Components to Bacterial Nanocellulose: A Comparison of Surface Qualities and Crystalline Properties

**DOI:** 10.4014/jmb.2106.06068

**Published:** 2021-07-23

**Authors:** Won Yeong Bang, Dong Hyun Kim, Mi Dan Kang, Jungwoo Yang, Taelin Huh, Young Woon Lim, Young Hoon Jung

**Affiliations:** 1 School of Food Science and Biotechnology, Kyungpook National University, Daegu 41566, Republic of Korea; 2 Ildong Bioscience, Pyeongtaek 17957, Republic of Korea; 3 Department of Biotechnology, Graduate School, Korea University, Seoul 02841, Republic of Korea; 4 School of Life Science and Biotechnology, Kyungpook National University, Daegu 41566, Republic of Korea; 5 School of Biological Sciences and Institution of Microbiology, Seoul National University, Seoul 08826, Republic of Korea

**Keywords:** Bacterial nanocellulose, *Komagataeibacter*, cellulose-binding protein, Avicel, carboxymethyl cellulose, fermentation

## Abstract

Bacterial nanocellulose (BNC) is a biocompatible material with a lot of potential. To make BNC commercially feasible, improvements in its production and surface qualities must be made. Here, we investigated the in situ fermentation and generation of BNC by addition of different cellulosic substrates such as Avicel and carboxymethylcellulose (CMC) and using *Komagataeibacter* sp. SFCB22-18. The addition of cellulosic substrates improved BNC production by a maximum of about 5 times and slightly modified its structural properties. The morphological and structural properties of BNC were investigated by using Fourier transform-infrared spectroscopy (FT-IR), scanning electron microscopy and X-ray diffraction. Furthermore, a type-A cellulose-binding protein derived from *Clostridium thermocellum*, C*t*CBD3, was used in a novel biological analytic approach to measure the surface crystallinity of the BNC. Because Avicel and CMC may adhere to microfibrils during BNC synthesis or crystallization, cellulose-binding protein could be a useful tool for identifying the crystalline properties of BNC with high sensitivity.

## Introduction

Cellulose, which is made up of a linear chain of glucose molecules linked by *β*-1,4-glycosidic linkages, is highly representative of renewable polymers in nature [1]. Cellulose is a biocompatible, biodegradable, and renewable substance that has received a great deal of attention, not only as a biopolymer capable of serving as an alternative to plastics in the chemical industry, but also as a medical material for tissue engineering and carrying drugs in the medical industry [2-6]. Furthermore, the derivatization of the cellulose surface can considerably improve its functionalities, broadening the applications of cellulose in numerous industries [7-10].

Lignocellulosic biomass is the most representative source of cellulose; however, because hemicellulose and lignin are present, severe and complex pretreatment steps with bleaching are required in order to achieve pure cellulose, resulting in high operating costs. As a result, microbial production of pure cellulose (*i.e.*, bacterial nanocellulose; BNC) has received a lot of attention as a potential alternative [2, 3, 11-13]. The use of bacteria to produce cellulose has a number of advantages, such as high purity and high specific surface area. For example, BNC has been widely utilized as a source of food, like Nata de coco, or as a food additive used for dietary fiber or as a thickener [14-16].

To enhance the applicability of BNC, significant cost reduction should be achieved by increasing BNC production as well as by specializing in the properties of BNC. There have been many studies aimed at obtaining BNC by using a novel superior microorganism or by optimizing fermentation conditions [17, 18]. Meanwhile, incorporating different additives including bio-derived polymers also showed beneficial effects on BNC production (Table 1) [19-25]. For example, carboxymethyl cellulose, a soluble form of cellulose, has generally provided an increase in BNC production yield, as well as modifying the cellulose properties, such as crystallinity. Meanwhile, various approaches to measuring the crystallinity of cellulose have been investigated, and recent studies showed that cellulose-binding protein (CBD) may become a more sensitive means of measuring crystallinity changes in cellulose [26, 27]. Since BNC is known to be one of the most crystalline polymers, measuring changes in the crystallinity of modified BNC could become a vital clue in proposing applications. To date, no studies have been investigating the crystallinity of BC using CBD.

In this study, we studied the effects of adding different cellulosic substrates on the synthesis and characteristics of BNC using *Komagataeibacter* sp. SFCB22-18 isolated from ripened persimmons [17]. The changes in various structural properties were investigated via scanning electron microscopy (SEM), Fourier-transform infrared (FT-IR) spectroscopy, X-ray diffraction (XRD), and the use of a type-A cellulose-binding protein derived from *Clostridium thermocellum* (C*t*CBD3). This study provides fundamental information on the effects of the addition of different types of cellulosic substrates during fermentation upon the production and structural modification of BNC.

## Materials and Methods

### Strain and Chemicals

*Komagataeibacter* sp. SFCB22-18, as obtained from ripened persimmons, was used in this study [17]. The strain was stored at -80°C in a 50% (v/v) glycerol solution. Carboxymethylcellulose (CMC) and Avicel were purchased from Sigma-Aldrich (USA). *Komagataeibacter* sp. SFCB22-18 was routinely cultivated in Hestrin–Schramm modified (HSM) medium containing 30 g glucose, 25 g yeast extract, 2.7 g Na_2_HPO_4_, 2.4 g acetic acid, and 5 g ethanol per liter. For bacterial cellulose (BC) production, an aliquot (1 ml) of the cell medium grown over 3 days was added to 100 ml of HSM medium in a 250-ml flask and incubated at 30°C for 7 days without shaking. For in situ production of BC, various concentrations, such as 0.1% (w/v) and 1% of CMC and Avicel, were added to the HSM medium.

### Production and Purification of BNC

BNC pellicles were produced and purified by following previous study [17] with slight modification. The pellicles floating on the surface of the culture medium were collected, immersed in 0.1-N NaOH at 80°C for 2 h to remove impurities such as bacteria cells and other medium components, and then washed with distilled water until the filtrate’s pH became neutral. The total solids content of the washed BC samples was measured by drying at 45°C for 72 h using a drying oven (BF-135C, BioFree, Korea). For further analysis, the BC was freeze-dried using a freeze dryer (Lyophilizer, IlShinBioBase Co., Korea) and stored at 4°C.

### Scanning Electron Microscopy (SEM) and Fourier Transform-Infrared (FT-IR) Spectroscopy Analysis

The lyophilized BNC samples were analyzed by SEM and FT-IR by following previous study [17] with slight modification.

SEM: The lyophilized BC samples were coated with a thin platinum film. The surface morphology of the BC samples was taken at an accelerating voltage of 16 kV using a field-emission scanning electron microscope (FE-SEM, SU8220; Hitachi, Japan).

FT-IR: The FT-IR spectra of lyophilized BC samples were analyzed using a Nicolet iS5 FT-IR Spectrometer (Thermo Scientific, USA) in the range of 4,000–450 cm^-1^ in the transmission mode. The spectra were recorded at a resolution of 4 cm^−1^ with 16 scans. The data were analyzed using OMNIC software (OMNIC v.9.7.46 firmware version 2.03; Thermo Scientific).

### X-Ray Diffractometer (XRD) Analysis

To investigate the crystallinity of the lyophilized BNC, X-ray diffractometer (D/Max-2500, Rigaku, Japan) analysis was performed using a cooper X-ray source. The diffracted radiation was measured in the range 2*θ* =5° to 50°, and the crystallinity index of cellulose, Crl, was calculated through the following equation [28]:

Crl (%) = [(I_200_-I_am_)/I_200_] × 100,

where I200 represents the total intensity (*i.e.*, crystalline region) at approximately 2*θ* = 22.7° and I_am_ represents the baseline intensity (*i.e.*, amorphous region) at approximately 2*θ* = 18°.

### Analysis of Protein Binding to the Crystalline Region of Cellulose

Quantitative analysis of the surface crystallinity of BNC was indirectly measured using C*t*CBD3 (UniProtKB: Q06851), a cellulose-binding protein derived from *C. thermocellum* (ATCC 27405), by following previous study [29] with slight modification. The C*t*CBD3 gene was cloned to *Escherichia coli* BL21(DE3) using the pET-21α. The strain harboring the C*t*CBD3 gene was grown in Luria-Bertani broth (BD, USA) containing 50 μg/ml of ampicillin at 37°C. When the optical density reached 0.5, isopropyl-β-D-thiogalactopyranoside (1 mM) was added and then incubated at 37°C for 4 h. After centrifugation at 10,000 ×*g* at 4°C for 30 min, re-suspended cells in 50 mM Tris-HCl (pH 8.0) were disrupted by sonication and the supernatant was centrifuged at 10,000 ×*g* at 4°C for 30 min. The recombinant enzyme was purified using Ni-NTA agarose (Qiagen, Germany) and 50 mM of imidazole. For analysis of binding affinity of C*t*CBD3 on BC samples, 5.6 nM of C*t*CBD3 was incubated with 1 mg of each BC sample in a 50 mM potassium phosphate buffer (pH 7.0) with a total reaction volume of 500 μl, for 12 h at 4°C. Subsequently, the reaction mixture was centrifuged at 13,000 ×*g* for 5 min to separate unbounded protein from the BC sample. Only unbound protein in the supernatant was quantified through the bicinchoninic acid assay, and the amount of bound protein was calculated by subtracting the amount of unbound protein from the total protein amount.

## Results and Discussion

### Effect on BNC Production

**Fig. 1:**
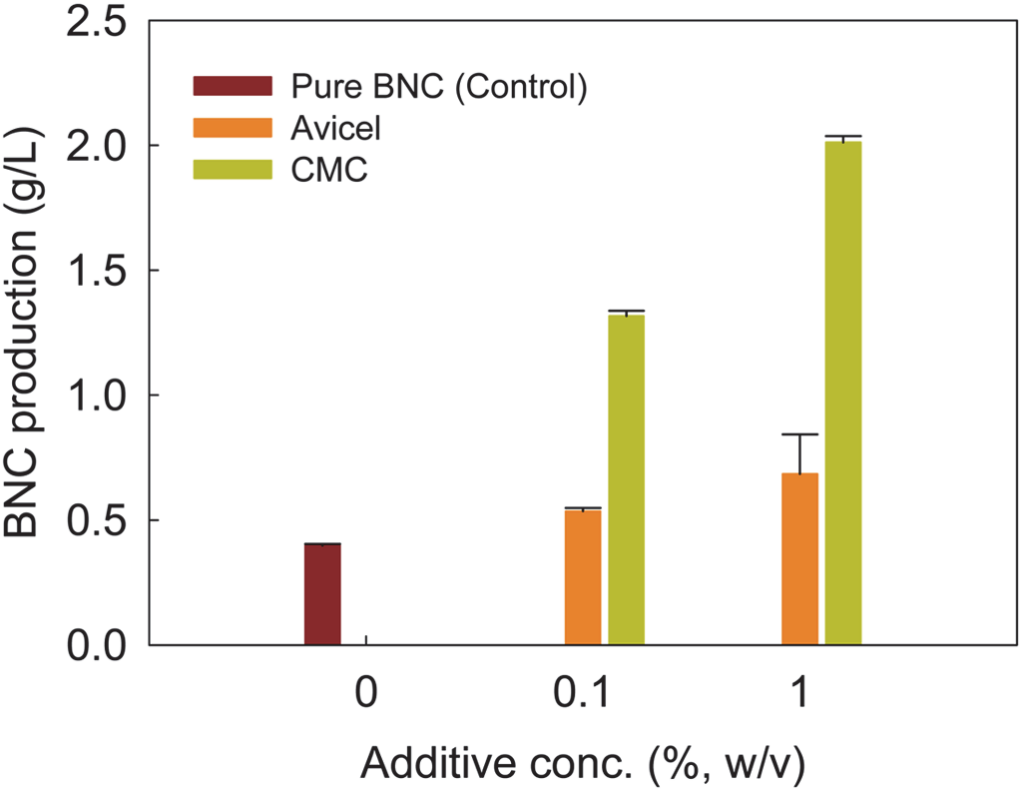
Production of bacterial nanocellulose by *Komagataeibacter* sp. SFCB22-18 in HSM medium containing different concentrations of Avicel and CMC. This experiment was performed in triplicate.

The impact of various cellulosic component additions upon BNC synthesis by *Komagataeibacter* sp. SFCB22-18 was initially examined. Because of the unique physical and chemical properties of Avicel (microcrystalline cellulose made from acid hydrolysis of wood pulp) and carboxymethylcellulose (CMC; cellulose derivatives with carboxymethyl groups at some of the glucopyranose hydroxyl groups), these celluloses are promising candidate materials for use in the food, pharmaceutical, paper, and cosmetic industries [30]. Thus, in this study Avicel and CMC were employed as cellulosic additives for altering BNC properties [31, 32]. Avicel and CMC addition showed slight increase in the BNC production (Fig. 1) when compared to the control group (*i.e.*, without additives, 0.4 g/l). With the additions of 0.1% Avicel and CMC, the *Komagataeibacter* strain produced 0.5 g/l and 0.7 g/l of cellulose pellicles, respectively. BNC production increased significantly when the quantities of cellulosic additions climbed to 1%. Particularly, the addition of 1% CMC resulted in the greatest BNC production of 2.0 g/l, which is equivalent to 5 times higher than in the control group. This increase might be because of the proper incorporation (or adsorption) of additives and reduction in crystallinity, which is a rate-limiting step during BNC production [31-33]. Meanwhile, Avicel did not show much improvement in BNC production because Avicel is less soluble than CMC [24]. Without optimization of reaction conditions, it was again proved that soluble additives may give more beneficial effects on production of BNC [31-33].

### Morphological and Structural Properties of Modified BNC

**Fig. 2:**
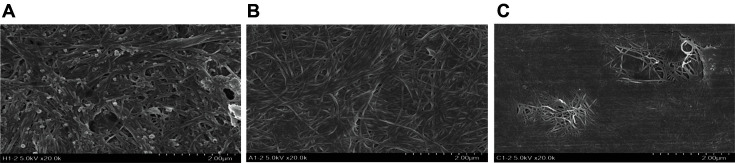
Scanning electron microscopy images of bacterial nanocellulose produced by *Komagataeibacter* sp. SFCB22-18 (**A**) without additives as a control; (**B**) with 1% (w/v) Avicel; and (**C**) with 1% (w/v) CMC.

**Fig. 3:**
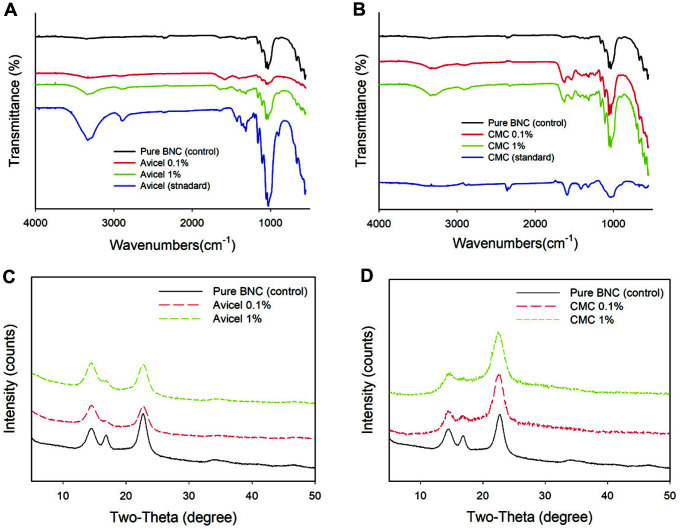
Fourier transform-infrared spectroscopy analysis and X-ray diffraction patterns of bacterial nanocellulose produced by *Komagataeibacter* sp. SFCB22-18 with (A, C) Avicel and (B, D) CMC.

SEM was used to examine the morphological structures of BNC with and without cellulosic additives (Fig. 2). In the pure BNC, thread-like, parallel stacked cellulose bundles with large clumps by aggregation were detected. However, the aggregates were not observable in modified BNC, which could be due to adsorption of additives on BCN surfaces [34-36]. This is most likely due to the constraints of intermolecular interactions between cellulose fibrils, including hydrogen bonding, the van der Waals force, and the electrostatic interactions, all of which help to stabilize the highly organized BC structure [37]. Accordingly, CMC-modified BNC had fibers that were somewhat longer than what Avicel-modified BNC had, probably due to repulsive force. Hence, BNC's structural morphology and crystalline characteristics were effectively altered by the incorporation of cellulosic additive. The FT-IR spectra (Figs. 3A-3B) of pure BNC and modified BNC revealed a broad OH peak stretching in the range of 3,500-3,000 cm^-1^ and C-O-C stretching at about 1,160 cm^-1^, as reported in a typical BNC spectrum [38, 39]. Additional peaks of 1,160 cm^-1^ (C-O-C stretching) and 1,035–1,060 cm^-1^ (C-O stretching) have been found in pure BNC. In Avicel- and CMC-altered BNC, the peak intensity at 3,500-3,000 cm^-1^ increased in comparison with that in pure BNC. In addition, in Avicel-altered BNC, the peak intensities at around 1,620–1,650 cm^-1^ (OH bending) increased, showing stronger adsorption of water molecules to BNC. This may be due to either the inhibition of BNC crystallization by reducing the degree of polymerization (DP) of the modified BNC [40] or the exposure of more OH groups by disruption of intermolecular interactions in BNC [41]. In CMC-modified BNC, strong absorption peaks at 1,572 cm^-1^, (*i.e.*, corresponding to the carboxyl group) [41] were well integrated (or adsorbed) on pure BNC.

### Degree of Crystallinity

The hardness, elasticity, permeability, and reactivity of cellulose may be indirectly reflected in its crystalline properties [42]. Because BNC is one of the most crystalline polymers, determining changes in the crystallinity of modified BNC could be a crucial indication in developing new applications. There has been no research on the crystallinity of BNC utilizing CBD yet. According to a prior study, crystallinity or BNC is negatively associated to polymerization of glucose [43]. As a result, we used XRD and crystalline cellulose-binding protein to examine and evaluate the crystalline characteristics of BNC modified by the inclusion of cellulosic additives.

XRD was used to determine the crystallinity of BNC after the various cellulosic components were added (Figs. 3C-3D). The XRD pattern of pure BNC revealed highly strong diffraction peaks at 2*θ* = 22.7° (primary peak), 14.5°, and 16.8°, corresponding to cellulose Iα, which is crystalline cellulose that is naturally formed [44]. The Avicel- and CMC-altered BNC showed similar patterns to cellulose I. However, as the concentrations of additives increased, the diffraction peaks at the 2*θ* angles of 16.8° decreased considerably in comparison with pure BNC. This means that the cellulose I structure was transformed to cellulose allomorph, resulting in the conversion of some crystalline structures into amorphous structures by the addition of cellulose components during BNC production [45, 46]. Furthermore, the addition of Avicel and CMC lowered the crystallinity index of BNC as evaluated by XRD (Table 1); for instance, while pure BNC had a crystallinity of 77.6%, modified BNC had a crystallinity of 69.2–73.4%. When the total crystallinity index (TCI, A1375/A2900 from FT-IR) was calculated after the addition of cellulosic substrates, a slight decrease in TCI values was detected. These findings are consistent with prior studies suggesting that the addition of cellulosic components slightly hinders the crystallization of BNC during synthesis, with the results of increased BNC production or water retention ability (*i.e.* OH group exposure at FT-IR).

**Fig. 4:**
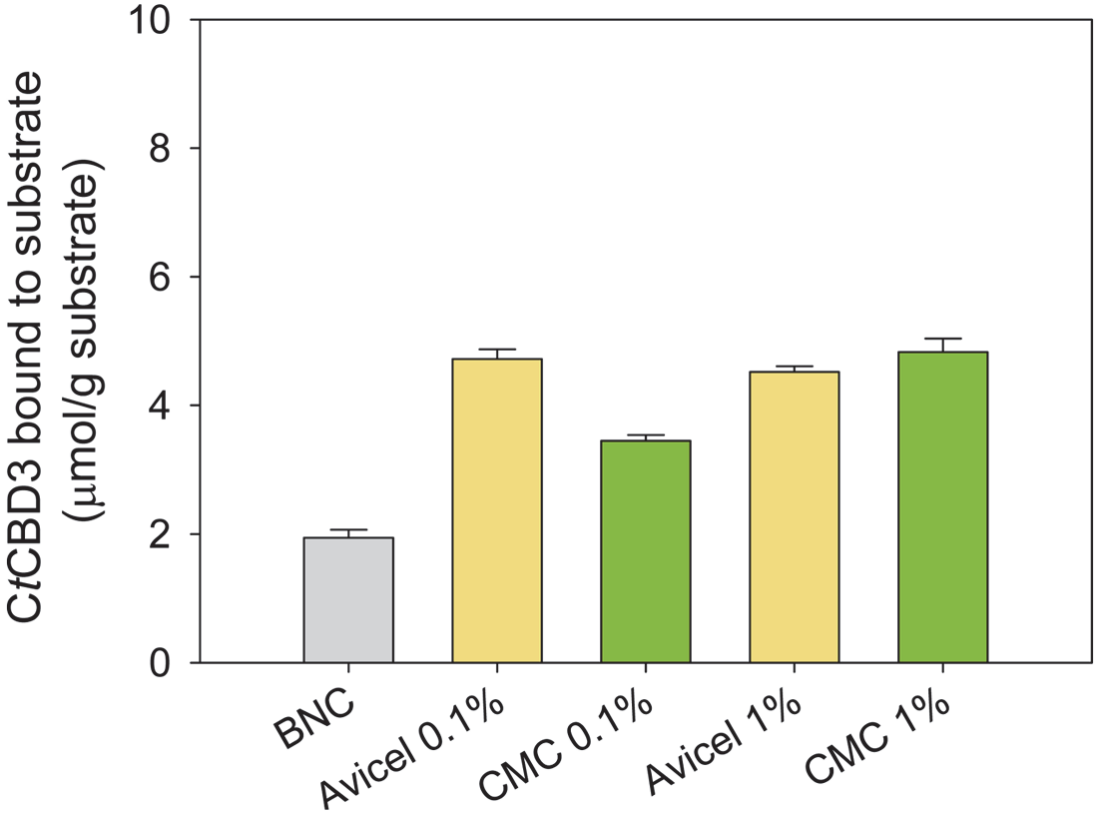
The binding ability of C*t*CBD3 to cellulose samples produced by *Komagataeibacter* sp. SFCB22-18 in an HSM medium containing different concentrations of Avicel and CMC. This experiment was performed in triplicate.

Next, using cellulose-binding protein, the surface accessibility of the crystalline region of BNC was observed. On the basis of the unique molecular recognition ability as to whether the binding region in cellulose is crystalline or amorphous, CBD has been proposed to understand the surface accessibility of cellulosic substrates [47, 48]. This is the first time that cellulose-binding proteins have been applied to elucidate the morphology of the BNC surface, one of the nano-sized cellulosic materials. Among cellulose-binding proteins, we selected C*t*CBD3, originating from *C. thermocellum* ATCC 27405 and belonging to type-A cellulose-binding proteins, which predominantly bind to the crystalline region of cellulose. The amount of bound protein in pure BNC was approximately 1.9 nmol/mg substrate (Fig. 4). The modified BNC samples showed 1.8–2.5 times higher binding affinities to C*t*CBD3 than pure BNC. Because cellulose-binding proteins are often bound to the surface of the cellulose, XRD or FT-IR focuses on the fiber's internal structure and bulk qualities [26], and the results may differ from XRD results. Therefore, the increased protein binding in this study indicates that cellulosic additives significantly exposed the crystalline areas of BNC to the surface of modified BNC. This could imply that distinct functional groups existed and were stable on the surface of modified BNC. As a result, adding cellulosic substrates like Avicel and CMC to the BNC during synthesis might effectively change the surface crystallinity of the BNC during fermentation. In summary, because Avicel and CMC may adhere to the surface of microfibrils during the formation or crystallization of BNC, determining the crystalline characteristics of BNC by evaluating binding qualities with cellulose-binding protein could be a viable option [36, 40, 49].

To summarize, cellulosic substrates with varying crystalline characteristics, such as Avicel and CMC, were utilized in this study to boost BNC synthesis and alter its surface crystallinity. XRD and the cellulose-binding protein were also used to compare the surface-crystalline characteristics of BNC. The addition of cellulosic substrates significantly improved the BNC production. Furthermore, it was found that the cellulose-binding protein could be used as part of a sensitive technique to measure the crystalline characteristics of BNC. The introduction of the binding protein may become a cost-effective and better alternative than the conventional method.

## Figures and Tables

**Table 1:** Literature review regarding bacterial nanocellulose incorporating different additives.

Strains	Additives	Concentrations	Target objectives	Ref.
*Gluconacetobacter xylinus* 53582	CMC, Pectin, Gelatin, Corn starch	1%, 3%, 5%	BC production	[19]
*G. xylinus* (BCRC 12335)	CMC	0-1%	Rehydration properties	[20]
*G. xylinus* (TISTR 975)	CNC	0%, 0.25%, 0.5%	Colloidal stability	[21]
*Acetobacter xylinum* x-2	Hydroxyapatite	1.5 times (v/v)	Artificial bones and scaffolds	[22]
*A. xylinum*	PVA	5%	Optical transparent films	[23]
*A. xylinum* (ATCC 700178)	CMC, Avicel, Sodium alginate, Agar	0.2%, 0.5%	BC production	[24]
*G. xylinus* (BRC- 5)	Chitosan	1%	Wound dressing	[25]

*PVA: Polyvinyl alcohol, CMC: Carboxymethyl cellulose, CNC: Cellulose nanocrystal

**Table 2:** Summary of the crystallinities of bacterial nanocellulose incorporating different concentrations of cellulosic additives.

		Crl (%)	TCI (%)	Relative value of CBM binding (%)

BC		77.6	0.994	100
Avicel		74.1	0.991	110.8
CMC		1.2	0.999	17.5

	Additive conc. (%)	Crl (%)	TCI (%)	Relative value of CBM binding (%)

BC + Avicel	0.1	72.2	0.993	243.3
	1.0	73.4	0.969	233.0
BC + CMC	0.1	69.2	0.988	177.8
	1.0	70.2	0.970	249.0

*BC: Bacterial nanocellulose, CMC: Carboxymethyl cellulose, CrI: Crystallinity index, TCI: Total crystallinity index, CBM: Cellulose binding Module
